# Syndrome latex-fruit: report of a case with cassava anaphylaxis

**DOI:** 10.1186/2045-7022-3-S3-P155

**Published:** 2013-07-25

**Authors:** ZM Almeida Sanchez, GL Hernandez Santana, E Rodriguez Plata, JA Martinez Tadeo, JC Garcia Robaina, C Gonzalez Colino

**Affiliations:** 1 Alergologia, Hospital Universitario Nuestra Señora de la Candelaria, Santa Cruz de Tenerife, Spain

## Background

Actually, there are few reports of cross-reactivity between cassava and latex in the syndrome latex-fruit. Of allergens in the latex are identified particularly relevant to this case, recall some of them: Hev b 6.02, major allergen, Hev b 1, structural homology to papain, and Hev b 5, which could be responsible for the cross reactivity between latex and cassava by their structural similarity with an allergen in cassava identified, known as Man and 5. Next we will detail the case of a patient with a diagnosis of "Syndrome latex-fruit allergy" and related to the group of fruits which been reported less frequently.

## Methods

A 32 year old woman reported that immediately after eating papaya presents cutaneus rash, oropharyngeal occupation; requires urgent medical attention. A year later, suffers two episodes of similar characteristics after eating avocado and cassava (the latter twice) and start referring oral pruritus chestnut intake. A directed anamnesis refers to several years after contact with latex oropharyngeal pruritus and rash presents urticarial in the contact area. It has good tolerance to banana, kiwi and tomato and free diet makes all the food involved in the reactions.

Tests were scheduled skin prick test (SPT) and prick-prick, and in vitro diagnostics.

## Results

The SPT were positive for latex, papaya, cassava, avocado and chestnut. Negative for the other studied food. Laboratory determinations provide us with high total IgE (289 IU / ml) with increased specific IgE to latex (91 KIU / L), avocado (0.84 KIU / L), chestnut (0.41 KIU / L) and tomato (6.20 KIU / L).

It also performs IgE immunodetection technique on yucca extract, yielding reactive bands IgE capable of setting different molecular weights (from 14 KDa to 75 KDa). The observed inhibition immunoblotting as latex extract can completely inhibit the binding of IgEs yucca extract, which shows that the patient's serum IgEs recognized epitopes yucca extract also present in latex and therefore the existence of cross-reactivity at a molecular level.

## Conclusion

A case of latex allergy syndrome-fruits with cross-reactivity between latex and different foods, some of them rare, like papaya and cassava. Both skin tests as "in vitro" are useful in our diagnosis, confirming the existence of reactive bands whose molecular weights may be compatible with the latex allergens and responsible for cross reactivity aforementioned (Hev b 1, Hev 6.02, Hev b 5).

## Disclosure of interest

None declared.

